# Use of Micellar Liquid Chromatography to Determine Mebendazole in Dairy Products and Breeding Waste from Bovine Animals

**DOI:** 10.3390/antibiotics9020086

**Published:** 2020-02-14

**Authors:** Rajendra Prasad Pawar, Pooja Mishra, Abhilasha Durgbanshi, Devasish Bose, Jaume Albiol-Chiva, Juan Peris-Vicente, Daniel García-Ferrer, Josep Esteve-Romero

**Affiliations:** 1 Department of Criminology and Forensic Science, Doctor Harisingh Gour Vishwavidyalaya (A Central University), Sagar, Madhya Pradesh 470003, India; rjforensics@gmail.com (R.P.P.); devonebose@gmail.com (D.B.); 2 Department of Chemistry, Doctor Harisingh Gour Vishwavidyalaya (A Central University), Sagar, Madhya Pradesh 470003, India; mishrapooja2013@gmail.com (P.M.); abhiasha126@gmail.com (A.D.); 3 Bioanalytical Chemistry, Department of Physical and Analytical Chemistry, ESTCE, Universitat Jaume I, 12071 Castelló, Spain; albiolj@uji.es; 4 Department of Analytical Chemistry, Faculty of Chemistry, Universitat de València, 46100 Burjassot, Spain; juan.peris@uv.es (J.P.-V.); danielgarciaferrer@hotmail.com (D.G.-F.)

**Keywords:** anthelmintic, micellar, food, milk, urine, validation

## Abstract

Mebendazole is an anthelmintic drug used in cattle production. However, residues may occur in produced food and in excretions, jeopardizing population health. A method based on micellar liquid chromatography (MLC) was developed to determine mebendazole in dairy products (milk, cheese, butter, and curd) and nitrogenous waste (urine and dung) from bovine animals. Sample treatment was expedited to simple dilution or solid-to-liquid extraction, followed by filtration and direct injection of the obtained solution. The analyte was resolved from matrix compounds in less than 8 min, using a C18 column and a mobile phase made up of 0.15 M sodium dodecyl sulfate (SDS)–6% 1-pentanol phosphate buffered at pH 7, and running at 1 mL/min under isocratic mode. Detection was performed by absorbance at 292 nm. The procedure was validated according to the guidelines of the EU Commission Decision 2002/657/EC in terms of: specificity, method calibration range (from the limit of quantification to 25–50 ppm), sensitivity (limit of detection 0.1–0.2 ppm; limit of quantification, 0.3–0.6 ppm), trueness (92.5–102.3%), precision (<7.5%, expressed at RSD), robustness, and stability. The method is reliable, sensitive, easy-to-handle, eco-friendly, safe, inexpensive, and provides a high sample-throughput. Therefore, it is useful for routine analysis as a screening or quantification method in a laboratory for drug-residue control.

## 1. Introduction

Livestock farming is now one of the most sought after agri-businesses mainly due to changes in dietary pattern and ease in export policy of raw and prepared food products. Poultry and cattle farming include the production of nutritious food such as egg, meat, milk, and other dairy products. Similar to other countries, in India, the rearing practices are done in traditional ways under natural conditions. However, in the current scenario, new breeds are also raised to gain more productivity and profitability [1]. Thus, there are many challenges faced by the producer, especially issues related to cattle health and hygiene. Infectious diseases have a great impact on livestock and they directly affect the productivity. The most prevalent infections among livestock are caused by helminths, of which liver flukes, lung worms, and tape worms are some of the common examples [2].

Thus, to cure these types of infections, drugs such as benzimidazoles, e.g., albendazole, fenbendazole, mebendazole (MBZ), etc., and macrocyclic lactones, e.g., ivermectin, are currently being widely used due to their broad-spectrum activity against helminths. There is a need to continuously develop new therapeutic agents, combinations of drugs, and treatments with increasing effect to cure and prevent these infections [3].

Another risk derived from veterinary treatments is the presence of residues of these drugs in animal derived food products, due to frequent dosing and inadequate withdrawal periods [4]. These drugs could be found as parent drug or as metabolite, which might adversely affect the end user. In humans, the risk groups are, in particular, children, pregnant women, and aged people because they have a weak defense mechanism [5]. Hypersensitivity, teratogenicity, mutagenicity, carcinogenicity, embryotoxicity, and decrease in the number of beneficial gastrointestinal bacteria are some of the reported adverse effects. Chronic exposure also leads to the growth of drug resistant parasites [6].

**Figure 1:**
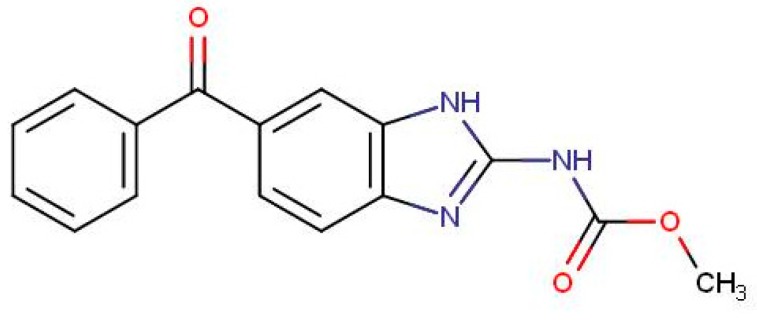
Structure of mebendazole.

With the increasing awareness of food and food products for human consumption and change in dietary pattern, attention is also paid to the risk to human health from food of animal origin. Maximum Residue Limits (MRLs) have been established by Food Safety and Standard Authority of India (FSSAI 2018), European Union (EU 2010), Food and Agriculture Organization of the United Nations (FAO)/ World Health Organization (WHO) (Codex Alimentarius Commission, 2015), and some other regulatory agencies for certain products of animal origin, such as milk, meat, etc. [7]. More specifically, the anthelmintic MBZ (Figure 1), for which the logarithm of the partition coefficient octanol/water (log Po/w) is 2.8 and the pKa of its protonated form is 6.6 [8], has been classified as “Not for use in animals from which milk is produced for human consumption” and thus should not be found in milk, milk-derived products, and farming wastes [9]. To ensure conformity with these regulations and provide a check on compliance with good veterinary practices, monitoring of residues of frequently used veterinary drugs in food products of animal origin is necessary [10]. For these reasons, there is always a need for a sensitive and selective analytical method, which could detect MBZ in these matrices [11].

Several research articles are available on the determination of veterinary drugs and their residues in animal derived food products. Most of the reported analytical methods use high performance liquid chromatography (HPLC) coupled with fixed wavelength ultraviolet [12,13], photo diode array [14] or mass spectrometry [15,16] detector.

On the other hand, classical analytical methods cannot meet the needs for the determination of these compounds in complex biological matrices. In the last decade, there has been an increase in the use of LC–tandem mass spectrometry (MS/MS), like LC–-time-of-flight mass spectrometry (LC-TOF) and LC–Orbitrap, which are versatile, highly selective, and sensitive techniques. Due to these advantages, LC-MS/MS is currently considered as the most suitable technique for simultaneous determination of veterinary drugs [17,18,19]. Nevertheless, it is an expensive technique that also requires expertise, laborious sample treatment steps, and expensive chemicals. In underdeveloped and developing countries such as India, availability of expensive techniques in routine analytical laboratory is limited. Thus, there is a need to develop and validate a simple method that is reliable, inexpensive, eco-friendly and able to determine drug residues in complex matrices, e.g., animal-derived food products to ensure food safety.

The aim of the research was the development of a method based on micellar liquid chromatography (MLC) to determine MBZ in milk and other dairy products such as curd, butter, and cheese to check the absence of residues in the food and ensure consumer protection, and farming waste (urine and dung), since its presence in excretions is proof the drug has been administered to the bovine animal. MLC allows direct injection of samples into the chromatographic system without any pretreatment; using sodium dodecyl sulfate (SDS) as a surfactant in mobile phase makes the method rapid, sensitive, cost effective, and eco-friendly, as well as serves as an alternative to conventional HPLC system [20,21,22,23]. These are some of the main reasons behind selecting MLC to carry out the present research work. The performance of the method was established by validation according to the guidelines of the EU Commission Decision 657/2002/EC [24]. Its suitability for routine analysis was checked by the analysis of incurred samples from local food stores and farms.

## 2. Materials and Methods

### 2.1. Chemical and Reagents

The analytical reference standards of mebendazole were purchased from Dr. Ehrenstorfer (Gmbh, Augsburg, Germany). SDS (99% purity), sodium dihydrogen phosphate, and sodium acetate (analytical grade) were obtained from Himedia Laboratories Private Limited (Mumbai, India). Hydrochloric acid (≈37%), sodium hydroxide (purity >99.0%), HPLC grade 1-propanol, 1-butanol, and 1-pentanol, were provided by Rankem, RFCL Limited (New Delhi, India). All solutions were filtered through 0.45-µm nylon membrane filters from Micron Separations Inc. (Westboro, MA, USA). Ultrapure water was in-lab elaborated from deionized water using an Ultrapure water generator device, Simplicity UV (Millipore S.A.S., Molsheim, France). This water was used to prepare the aqueous solutions.

An ultrasonic bath (Model Ultrasons-H; Selecta, Barcelona, Spain) was used to complete the solubilization of the solids and ultrasonicate the solutions. The pH of the mobile phase was measured by using a digital pH meter pH-102/103 Contech, Instruments Limited (Mumbai, India). Analytical weighing scale was from Mettler Toledo India Private Limited (Mumbai, India).

### 2.2. Preparation of Solutions

Micellar solutions were prepared by accurately weighing an adequate amount of SDS and sodium dihydrogen phosphate or sodium acetate salt, which were then dissolved in ultrapure water with a magnetic stirrer. Adjustment of the pH was performed by the addition of drops of NaOH (0.1 M) or HCl (0.1 M). Afterwards, the organic solvent was introduced to attain the desired proportion, and the volumetric flask was filled with water. Finally, the solution was ultrasonicated for 5 min, and filtered through a 0.45-μm nylon membrane filter with the aid of a vacuum pump.

Stock solution of mebendazole (100 mg/L) was prepared by dissolving 1 mg of standard compound in 10 mL of methanol. This solution was sonicated for 5 min in an ultrasonic bath for the proper dissolution of the compound and finally stored in 10 mL amber volumetric flask at 4 °C. Working solutions were prepared from the stock solution by the proper dilution in mobile phase, and renewed each month.

### 2.3. Chromatographic Conditions

Chromatograph was a Shimadzu Prominence HPLC System, Shimadzu Corporation (Kyoto, Japan) equipped with an isocratic pump LC-20 AT, an autosampler SIL-20AC, and a diode array detector SPD-M20 A (190–800 nm). The column was a SPHER-100 C18 100A (250 mm × 4.6 mm × 5 µm particle size) from Princeton Chromatography INC (Cranbury, NJ, USA). The mobile phase was an aqueous solution of 0.15 M SDS–6% 1-pentanol–0.01 M Na_2_HPO_4_ buffered at pH 7, running at 1 mL/min under isocratic mode. Injection volume and absorbance detection wavelength were 20 μL and 292 nm, respectively. All injected solutions were previously filtered through 0.45-μm nylon membrane filters by manual pushing with a 3-mL syringe. Processed samples were discarded after injection. The specific working procedure required for the HPLC system when using micellar mobile phases was previously reported by Pooja Mishra et al. [25].

The control of the instrumentation and the registration of the signal were performed through the software Shimadzu LC Solution software version 1.22 SP1. The main chromatographic parameters—retention time (*t*_R_), retention factor (k), efficiency (number of theoretical plates, N), and asymmetry (T)—were calculated as indicated in [26].

### 2.4. Sample Collection and Processing

Milk, curd, butter, and cheese samples were collected from dairy located at Sagar and Chhindwara city of Madhya Pradesh, India. Milk samples were also provided by the milkman. Subsequently, dung and urine samples were collected from bovine animals, which were medically treated with MBZ. Blank samples were collected from the bovine animal having no previous history of any kind of treatment. Blank samples were also treated as incurred ones. Samples were stored at −20 °C.

Milk and urine samples were 1/10 and 1/5, respectively, diluted with mobile phase, before injecting the samples into the chromatographic system [27]. A batch stirring-assisted solid-to-liquid extraction (BSASLE) followed by a batch ultrasound-assisted solid-to-liquid extraction (BUASLE) [28] procedure was used to obtain the analyte from curd, butter milk, butter, cheese, and dung. These samples were finely ground using a mincer (Model MZ10, Petra Electric, Burgau, Germany) at 5000 rpm for 5 min. Five grams of the solid sample were mixed with 20 mL of mobile phase in an Erlenmeyer flask. The solution was shaken in a magnetic stirrer for 1 h and ultrasonicated for 15 min, filtered and filled up to 25 mL. Fortification was performed as follows: a known amount of MBZ solution was injected into the weighed sample, which was left for one day to achieve integration of the analyte into the sample. These spiked samples imitate those “naturally” contaminated [29]. Finally, they were treated as the unfortified samples.

Before injection, refrigerated solutions were thawed at room temperature to solve the SDS crystals formed overnight.

Regarding the units used to quantify in samples, “ppm” refers to mg/L in liquid samples (milk and urine), while “mg/kg” in solid ones (curd, cheese, butter, and dung).

This study was approved by the University Jaume I (UJI-QBA-063-2019).

### 2.5. Statistical Calculations for Calibration Evaluation

The statistical calculations were performed as follows.

Homoscedasticity of the residuals in the calibration model [30]: each standard solution was injected three times and the variances were calculated. We performed a Snedecor’s F distribution test by comparing the highest and the lowest variance.

Relative residual standard deviation [26]: This value was the standard deviation of the residuals (obtained by the least-square regression method) divided by the slope and divided by the average of the concentration of all the points included in the curve.

*t*-Student test on r significant [30]: The value of *t*_exp_ of r was calculated by the following formula:

$$t_{exp}=\frac{ABS\left(r\right)\sqrt{n-2}}{\sqrt{1-r^{2}}}$$

where ABS means absolute value, n is the number of points in the calibration curve, and r is the correlation coefficient. This was compared to the *t*-Students tabulated value for *n*-2 degrees of freedom, a significance level of 5%, and two sides. If *t*_exp_ > *t*_tabulated_, there was a significant correlation between x and y.

Squared Cook distance (CD^2^) [30]: This parameter was calculated for each point by the following formula:

$$CD_{i}^{2}=\frac{\sum_{j=1}^{n}{\left({\hat{y}}_{j}-{\hat{y}}_{j\left(i\right)}\right)}^{2}}{2s_{y/x}}$$

where ${\hat{y}}_{j}$ is the predicted value for y the point j, ${\hat{y}}_{j\left(i\right)}$ is the predicted value for y for the point *j*, in the curve constructed by removing the point i, and *s*_y/x_ is the standard deviation of the residuals provided by the curve with all the points. An *i* point with a CD^2^ larger than 1 was rejected.

Standardized residuals [30]: This is the quotient between the value of the residual and *s*_y/x_. A point with a value >3 was rejected.

## 3. Results and Discussion

### 3.1. Optimization of the Chromatographic Conditions

It has been largely proven that a C18 column, SDS as surfactant, a flow rate of 1 mL/min under isocratic mode, and an injection volume of 20 μL are the most adequate conditions to determine drugs by MLC [22]. In the present work, we aimed to optimize specific parameters such as organic solvent nature and proportion, SDS concentration, and pH in the mobile phase, and the detection conditions. Assays were performed using a standard solution containing 0.5 mg/L of MBZ.

#### 3.1.1. Optimization of the Detection Conditions

MBZ is a UV-active compound due to the presence of conjugated saturated and unsaturated bonds as well as the presence of heteroatoms such as nitrogen and oxygen (Figure 1), which increase its sensitivity in UV region. The reported λ max for MBZ is 292 nm. Under the optimal conditions, the UV absorbance spectrum of MBZ was taken during the chromatographic run at the retention time, and the wavelength with the highest signal-to-noise ratio was found at the same wavelength. Thus, 292 nm was selected as the optimum wavelength for MBZ.

#### 3.1.2. Optimization of Surfactant Concentration

In MLC, the first step towards mobile phase optimization is the selection of the proper SDS concentration. Three different concentrations of SDS, i.e., 0.05, 0.10, and 0.15 M, were selected, as 0.05–0.15 is the typically recommended working interval for SDS concentration in MLC [22]. Using the concentration of SDS mentioned above, chromatographic parameters such as capacity factor (14.2, 9.5, and 7.3, respectively), efficiency (982, 742, and 651, respectively), and asymmetry factor (1.6, 1.4, and 1.4, respectively) were compared.

Comparison of retention time in pure micellar mobile phase revealed that, with the increase in concentration of SDS, the retention time of MBZ decreases. Over the critical micellar concentration, the augmentation of the analytical concentration of SDS results in an increase in the number of micelles, whereas the concentration of free monomer and the number of monomers coated on the stationary phase remain nearly invariant. Since MBZ is neutral and hydrophobic, it spends more time with the hydrophobic core of the micelles and therefore, as the concentration of SDS increases, the retention time of MBZ decreases. Thus, 0.15 M was chosen as the optimum SDS concentration for further analysis.

#### 3.1.3. Optimization of pH

After optimizing the SDS concentration, the next step was the selection of pH. Thus, to optimize the chromatographic behavior of MBZ, three pH levels, i.e., pH 3, 5, and 7, were studied. According to the pKa and the structure of MBZ, at pH 3 and 5, the analyte is monocationic, while neutral at pH 7. Otherwise, the substance is relatively hydrophobic. Results of the chromatographic elution were: capacity factor of 10.5, 9.7, and 7.3, respectively; efficiency of 454, 498, and 651, respectively; and asymmetry factor of 1.8, 1.7, and 1.4, respectively.

Since SDS is negatively charged, SDS monomers modified stationary will carry a negative charge. Therefore, due to electrostatic attraction, the positively charged MBZ molecule will be attracted to the negatively charged (modified) stationary phase, resulting in a higher retention time. As the pH increases to 7, the protonation of amino group decreases and the molecule becomes neutral and hydrophobic. Due to this change in polarity, the interaction of the molecule with the stationary phase decreases and the molecule shows more interaction with the hydrophobic core of the SDS micelle. This results in decreased retention of MBZ at higher pH. Hence, pH 7 was selected as an optimum pH for further analysis. This pH is also known for extending the column life, which was another reason for selecting the pH 7. To maintain the pH during the chromatographic run, 0.01 M sodium dihydrogen phosphate buffer salt was added to the mobile phase.

#### 3.1.4. Optimization of the Organic Solvent

**Figure 2:**
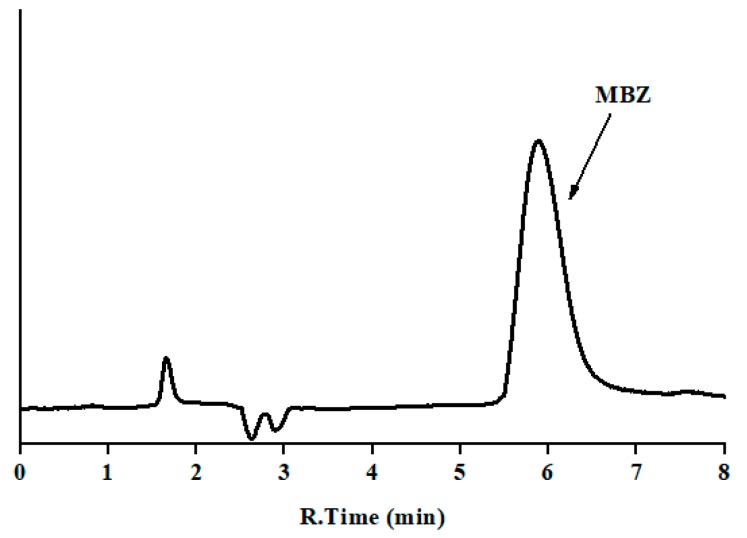
Chromatogram of standard mebendazole: mobile phase 0.15 M sodium dodecyl sulfate (SDS), 6% 1-pentanol (*v*/*v*) at pH 7; flow rate 1.0 mL/min; injection volume 20 μL; and detection at 292 nm (y-axis is the absorbance in arbitrary units, and the same scale applies for Figure 2, Figure 3 and Figure 4).

Another important factor which was considered here is the log Po/w of MBZ, which is 2.8, indicating that it is a non-polar compound that could result in longer interaction with the core of micelle. These types of compounds do not separate in the desirable time period using pure micellar mobile phases. Thus, to improve the peak efficiency and asymmetry, addition of organic modifier to pure micellar mobile phase is sometimes desirable. Due to higher elution strength, 1-propanol, 1-butanol, and 1-pentanol could be used in the micellar mobile phase, which helps to reduce the retention time and improve the peak shape. Thus, to select an appropriate organic modifier, MBZ was injected using a mobile phase having maximum concentration of SDS, i.e., 0.15 M, and with varying concentration of organic modifier of different chain length, i.e., 0.15 M SDS and 12% propanol (*v*/*v*), 8% butanol (*v*/*v*), or 6% 1-pentanol (*v*/*v*) at pH 7. The obtained results for the chromatographic parameters were: capacity factor of 8.3, 5.0, and 2.7, respectively; efficiency of 1982, 2251, and 2584, respectively; and asymmetry factor of 1.2 in all cases. We can observe that 1-pentanol decreases the analysis time to less than 10 min and improves the peak profile of the compound (Figure 2), if compared to the other alcohols. Thus, based on the above-mentioned chromatographic behavior, it was decided to carry out further analysis using 0.15 M SDS with 6% 1-pentanol buffered to pH 7 with 0.01 M sodium dihydrogen phosphate buffer salt.

A system suitability test (SST) was performed under the optimal work conditions, by analyzing a standard solution of 0.5 mg/L of MBZ six consecutive times. The results for tested parameters (acceptance criteria) are as follows: *t*_0_ = 1.6 min; *t*_R_ (min), 5.88 ± 0.05; RSD of *t*_R_, 0.9% (<1.0%); RSD of peak area, 0.8% (<1.0%); RSD of width at half-height, 0.9% (<1.0%); retention factor, 2.7 (>2); efficiency, 2584 theoretical plates (>2000); and asymmetry, 1.2 (0.8–1.6). The entire absorption spectrum was taken at the maximum, and leading and tailing edge at half- and 5%-height for peak purity studies.

### 3.2. Method Validation

The procedure was validated according to the guidelines of the EU Commission Decision 2002/657/EC, which was specifically elaborated to determine organic residues in foodstuff [24] and other documents about validation [26,30,31] in terms of: instrumental calibration range and sensitivity, robustness (in standard solution), specificity, method calibration range, sensitivity, trueness, precision (in matrix), and stability. Unless specified, the statistical tests (Snedecor’s F distribution test and *t*-Student test on r significant) were performed at a significance level (α) of 5%.

#### 3.2.1. Instrumental Calibration Range and Sensitivity

Nine standard solutions were prepared in the range 0.06–5 mg/L, by triplicate. The y-variances were found significantly equal along the studied concentrations by a Snedecor’s F distribution test; thus, the residuals were considered homoscedastic. The calibration curve was plotted using the peak area vs. concentration. The slope (53,400 ± 800) (L/mg) and intercept (1000 ± 400) were determined by the least square linear regression method. The response of the tested compounds was linear (r^2^ = 0.9996; relative residual standard deviation <1.5%; *t*-Student test on *r* significant). Residual distribution was found normal, by visualization of the plot residuals vs. concentration. No outliers were detected, since the squared Cook distances were <1 and the standardized residuals were <3.

After the linearity study, limit of detection (LOD) and limit of quantification (LOQ) were calculated. The LOD was determined using 3.3 s criterion, as 3.3 is the standard deviation of the y-intercept divided by the slope of the calibration curve, which was found to be 0.02 mg/L. Similarly, the limit of quantification (LOQ) was determined using the 10 s criterion and was found to be 0.06 mg/L. The upper limit of quantification (ULOQ) was set to 5 mg/L.

#### 3.2.2. Ruggedness

Ruggedness of the procedure was investigated by introducing small deliberated changes into the main chromatographic parameters, such as concentration of SDS, percentage of 1-pentanol (*v*/*v*), pH, and the flow rate of the mobile phase, and evaluating the variation of retention time and peak area by a one-fact-at-a-time approach. The study was performed by injecting standard solution of 0.5 mg/L. Studied intervals and results can be seen in Table 1.

Variation in the flow rate had a higher influence on the retention time of the analytes, as expected. However, this experimental condition is well-controlled by the instrumentation and hardly ever undergoes modifications. The other factors were barely affected (<4%) by the chromatographic parameters, thus the method is robust enough to face the usual oscillations of the experimental conditions that may occur during the usual laboratory work.

#### 3.2.3. Specificity

**Figure 3:**
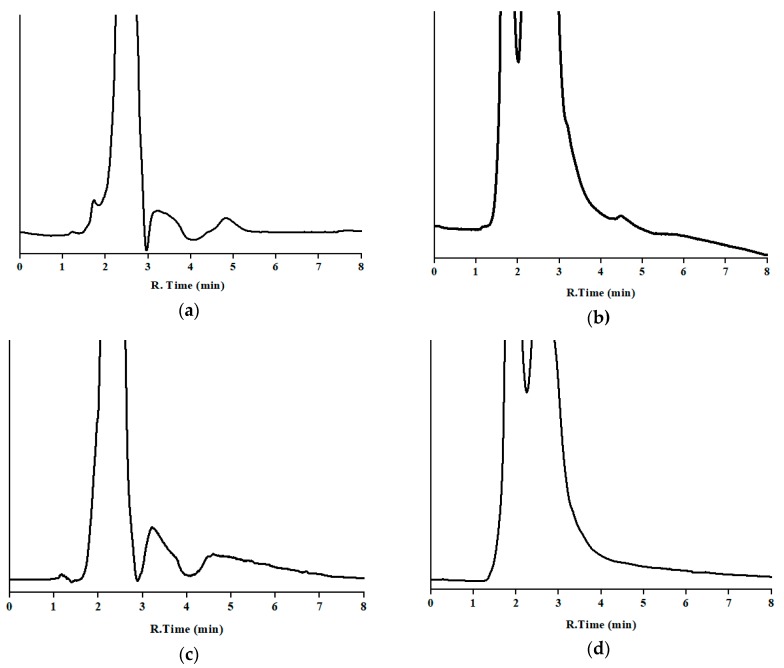

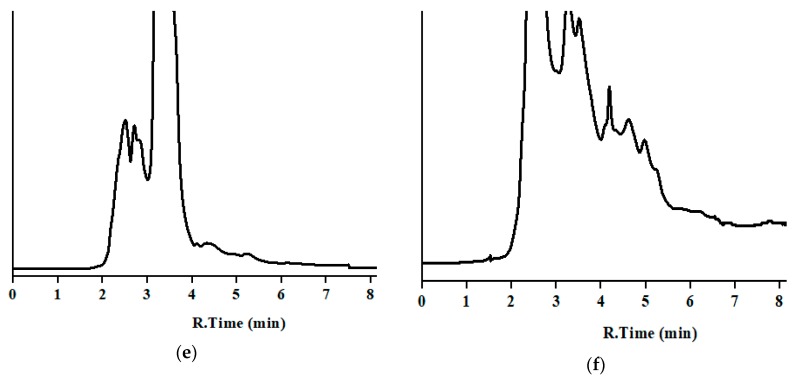
Chromatograms of blank samples in the optimum chromatographic conditions: (**a**) milk; (**b**) cheese; (**c**) butter; (**d**) curd; (**e**) urine; and (**f**) dung (y-axis is the absorbance in arbitrary units, and the same scale applies for Figure 2, Figure 3 and Figure 4).

To study the selectivity of the developed method, for each studied food or waste, one blank sample was analyzed before (Figure 3) and after being fortified at 2.5 ppm of MBZ, except milk, which was spiked at 5 ppm. In all cases, the injected solution contained 0.5 mg/L of the drug.

Blank samples exhibited several broad peaks from the dead time to nearly 5 min, and then enough distance to avoid overlapping with the analyte, and a quite stable baseline afterwards. No peak was observed at or close to the window time of the drug. Regarding the fortified samples, chromatograms showed similar shape to the corresponding blanks, plus the peak of MBZ, which displayed similar retention time, shape, and area as that obtained by the analysis of a standard solution (see Section 3.1.4). No partial overlapping was noticed. A peak purity test was performed. In the fortified samples, UV absorbance spectra were taken in the chromatograms from spiked samples at the same points as indicated in Section 3.1.4, and compared by visual observation by overlaying those obtained by the analysis of the working standard solution, and no significant difference was noticed. Therefore, no noteworthy interference was found by either the endogenous compounds or the front of the chromatogram.

#### 3.2.4. Method Calibration Range

Method limit of detection (MLOD), limit of quantification (MLOQ), and upper limit of quantification (MULOQ) were calculated from LOD, LOQ, and ULOQ, considering the pretreatment of the sample. Results are as follows: for milk, 0.2, 0.6, and 50 ppm, respectively; and for urine, cheese, butter, curd, and dung, 0.1, 0.3, and 25 ppm, respectively.

#### 3.2.5. Trueness and Precision

These parameters were determined for each sample at three levels (1, 2.5, and 5 ppm) using fortified samples.

Trueness was determined by the analysis of six samples as the quotient between the found concentration and the true one. Repeatability was calculated from the same experiments as the relative standard deviation of the found concentrations. Intermediate precision was measured by carrying out the previous procedure five times on different days (using renewed samples), over a three-month period, as the relative standard deviation of the five obtained concentrations. Results can be seen in Table 2. Bias (92.5–102.3%) and variability (<7.5%) of the results comply with the acceptance criteria of the guide (80–110% and <8.4%, respectively), indicating the reliability of the quantitative information provided by the method.

#### 3.2.6. Stability

The possible degradation of the analyte was examined in standard solution and in matrix. Assayed conditions were those likely to apply during the usual storage, handling, and analysis. Stability was established by the monitoring of ratio found concentration at different times divided by the peak area and the visual observation of other peaks from decomposition products. Decay was considered significant if the peak area dropped >5% for standard solution and >15% in matrix.

Tests were conducted as described below. In all cases, no decay peaks were noticed, and the variation of the concentration was inside the acceptance criteria. Therefore, MBZ was found quite stable during the studied period.

(a) Standard solution: A working standard solution of 0.5 mg/L was analyzed (Day 0) and kept in amber vial in the fridge at 4 °C. Everything three days for one month, the solution was thawed, an aliquot taken and analyzed, and then returned to the fridge. Consequently, working standard solution can be used at the laboratory for analysis purposes without introducing a bias in the measures during this period.
(b) In samples (this protocol was applied for each studied matrix): Twenty-one fresh blank samples were spiked with the required amount to obtain an injected solution of 0.5 mg/L. One of them was immediately analyzed, and the others were kept in a freezer at −20 °C. These were analyzed weekly. Therefore, samples can be analyzed until this period after reception, without affecting the trustworthiness of the result.

### 3.3. Analysis of Incurred Samples

**Figure 4:**
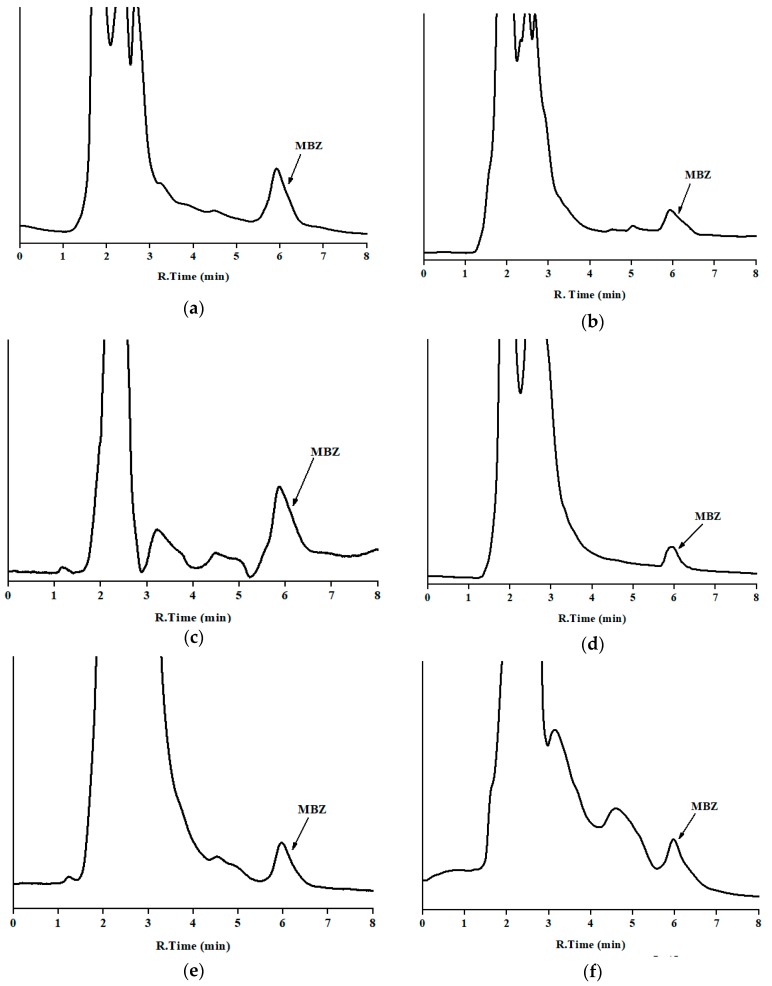
Chromatograms of incurred samples in the optimum chromatographic conditions: (**a**) milk; (**b**) cheese; (**c**) butter; (**d**) curd; (**e**) urine; and (**f**) dung (y-axis is the absorbance in arbitrary units, and the same scale applies for Figure 2, Figure 3 and Figure 4). Corresponding concentration are in Table 3 (set 1).

The developed method was applied to analyze MBZ in 30 incurred samples, five per analyzed sample. MBZ was clearly identified and quantified (Table 3) without interferences for matrices of milk, curd, butter, and cheese as well as dung and urine samples. The low detection limit of the developed method is useful for the determination of MBZ, which is prohibited in both milk and milk products. Concentration in sample were in the lower zone of the calibration curve, near the LOQ. Chromatograms of incurred samples of milk, milk products, and nitrogenous waste of the bovine animal can be seen in Figure 4.

As far as we know, this is the first article describing a method to determine mebendazole in cheese, butter, curd, dung, and urine using liquid chromatography coupled to absorbance detection. Using this technique, MBZ has been quantified in milk using complex extraction procedures (trueness 82.3–105.2% and precision <13.4%) [32] or specific extraction devices (trueness 70.2–117.6% and precision <10.9%) [33]. Those methods exhibit lower analytical performances, are more expensive and use larger quantity of toxic chemicals. According to our knowledge, this is the first article using direct injection to analyze MBZ in milk.

## 4. Conclusions

The paper herein describes an eco-friendly, fast, and simple analytical method for the determination of MBZ in different dairy products, such as milk, curd, cheese, butter, etc. The method was developed and validated in a single laboratory according to an international guideline. Good values for the main validation parameters such as linearity, limit of detection, limit of quantification, trueness, precision, ruggedness, etc. were obtained, indicating the suitability of this method for the determination of MBZ in different edible products derived from bovine animal, which are regarded as complex biological matrices. Efforts were made to determine MBZ in the nitrogenous waste (dung and urine) of the bovine animal as well. MLC allows rapid determination of the MBZ by direct injection of the samples except filtration.

It can be used in routine analysis for drug-residue control purposes in food quality control or safety laboratories, as either screening or quantification method in food products from bovine animals where MBZ is suspected to limit the risk for the population. It is also useful to determine the drug in bovine nitrogenous waste, to monitor the use in farming. It worth mentioning that the use of MBZ is not recommended for bovine animals. Another feature of the developed method is that it can be considered as an eco-friendly method because the SDS used throughout the experiments is biodegradable and a very low amount of organic solvents is added when compared to conventional HPLC methods. Besides, it is easy-to-conduct, due to the simplicity of the sample preparation, and thus many samples can be analyzed in a short period of time, resulting in a high sample throughput. Therefore, it can also be useful for examination of such complex biological samples in forensic science laboratories or chemical examiner laboratories.

## Figures and Tables

**Table 1:** Robustness study of the developed method (RSD, relative standard deviation).

Parameter	Level	RSD of Retention Time (%)	RSD of Peak Area (%)
SDS Concentration (M)	0.14–0.16	3.1	2.5
1-pentanol (%, *v*/*v*)	5.75–6.25	2.3	1.5
pH	6.9–7.1	1.2	2.6
Flow rate (mL/min)	0.9–1.1	9.8	2.0

**Table 2:** Trueness and precision of the method (results for 1, 2.5, and 5 ppm).

Matrix	Repeatability ^a^ (RSD, %)	Intermediate Precision ^b^ (RSD, %)	Trueness ^a^ (%)
Milk	(3.4; 2.3; 2.7)	(3.7; 4.8; 2.2)	(98.5; 99.1; 99.8)
Cheese	(3.2; 3.5; 2.9)	(5.1; 4.3; 3.9)	(97.3; 99.3;99.8)
Butter	(4.0; 5.2; 3.7)	(4.8; 4.0; 3.5)	(92.5; 94.3; 96.1)
Curd	(4.6; 3.9; 2.6)	(5.4; 4.3; 3.5)	(97.7; 101.6; 97.8)
Urine	(2.9; 2.7; 3.5)	(3.2; 3.1; 2.8)	(96.8; 99.4; 102.3)
Dung	(5.2; 4.6; 4.0)	(7.5; 6.7; 5.1)	(96.7; 97.3; 99.7)

^a^*n* = 6; ^b^ *n* = 5; RSD, relative standard deviation.

**Table 3:** Concentration of MBZ (ppm) found in different incurred samples.

Kind of Sample	Set 1	Set 2	Set 3	Set 4	Set 5
Milk	4.94	2.1	1.5	7.4	1.0
Curd	0.30	n.d.	n.d.	1.8	n.d.
Cheese	6.70	4.2	2.7	9.5	1.2
Butter	1.23	0.9	0.5	4.1	n.d.
Dung	3.14	1.5	0.9	5.9	0.7
Urine	2.96	2.3	0.6	4.1	1.2
